# Impact of Breastfeeding on Low Birthweight Infants, Weight Disorders in Infants, and Child Development

**DOI:** 10.7759/cureus.32894

**Published:** 2022-12-24

**Authors:** Hanaa Juharji, Khalid Albalawi, Mohammed Aldwaighri, Ahmed Almalki, Hisham Alshiti, Wahhaj Kattan, Mohammed Alqarni, Sulaiman Alsulaimani, Tuqa AlShaikh, Feras Alsulaimani

**Affiliations:** 1 Family Medicine, Makkah Healthcare Cluster, Jeddah, SAU; 2 College of Medicine, King Abdulaziz University, Jeddah, SAU; 3 College of Medicine, King Saud University, Riyadh, SAU; 4 Public Health, Ministry of Health, Dammam, SAU; 5 Internal Medicine, Heraa General Hospital, Makkah, SAU

**Keywords:** neonate, mortality, preterm, nutrition, breast milk

## Abstract

Infancy has been proven as the best time to improve health outcomes for the later stage of life. The composition of human breast milk has evolved over millennia to support and maintain the infant's life during the early years of life. To achieve life-sustaining effects, human breast milk is packed with fats, proteins, carbohydrates, and a wide range of bioactive compounds such as immunoglobulins, lactoferrin, and cytokines. The immunological compounds in breast milk have been shown to curtail gastrointestinal tract infections, respiratory tract infections, hospital admissions, acute otitis media, allergic reactions, and urinary tract infections. Although breastfeeding causes newborns to gain less weight at the beginning of their lives than formula milk does, breast milk improves body composition by low adiposity. A higher adipose deposition in infants is linked with an increased risk of child obesity in the future. Due to significant health benefits, the World Health Organization (WHO) recommends initiating breastfeeding within one hour after birth and continuing for at least six months. Breastfeeding has emerged as a superior source of nutrition that can promote healthy physiological and cognitive development and protect against disease challenges in low birthweight infants. This review summarizes potential evidence that highlights the potential health impact of breast milk in low birthweight infants.

## Introduction and background

Breastmilk is a nutrient-enriched biofluid that has evolved over millions of years to optimize the nutritional requirements of infants [1]. The composition of breast milk consists of complex proteins, lipids, carbohydrates, and biologically active compounds such as nucleotides and immunoglobulins [2]. To impart early health benefits, breastfeeding infants is regarded as one of the most important care processes that improve survival, developmental, and health outcomes. The findings of a meta-analysis by Victoria et al. on breastfeeding revealed that it has a protective effect against childhood infections including pneumonia and diarrhea, improves cognitive development, and reduces premature mortality in infants [3]. As low birthweight (LBW) infants are more vulnerable, health professionals recommend exclusive breastfeeding as the primary nutritional program on which their care should be based [4]. For this reason, breastfeeding is an important research subject that needs to be explored in various dimensions, to develop empirical support for practices that breastfeeding mothers can use to care for their preterm infants, especially the first six months which are critical to the infant’s survival [5]. In this article, the authors review and discuss the most recent evidence in the literature on the effect of breastfeeding on infections, weight gain, and child development with a focus on LBW infants.

Despite significant evidence of the health benefits of breastfeeding, many women in high-income countries fail to achieve this metric. Sriraman attributes such behaviors to low knowledge among physicians that fail to forward appropriate recommendations [6]. The functional anatomy of the breast and the breastfeeding process is of great importance because it traces the synthesis of milk to the action of the glandular luminal cells. In essence, the induction of the secretory process is done by prolactin hormone at the time of postpartum withdrawal [7]. Given the important role that hormones, for example, prolactin, play in the physiology of lactation, clinicians need to understand the hormonal dynamics and stimulus in each of the three stages of lactogenesis [8].

Children who are born with complications, such as LBW infants, are particularly vulnerable to developmental conditions because of the underlying implications of defects that they were born with [9]. According to Sriraman, breastmilk is the most functional nutritional provision that infants need for healthy development. However, physicians often engage in the treatment of conditions that can be controlled by improving and supporting the healthy development of infants. This is the empirical basis that Sriraman uses to emphasize the importance of breastmilk as individualized medicine for the LBW infant. In this recent study in which the value of breastmilk is contextualized, Sriraman highlights trends in breastfeeding practices among mothers in the United States that provide even greater context for modeling better interventions and health training [6]. To ensure that breastmilk is used to meet the nutritional needs of LBW infants, there is a need to sensitize mothers to practice breastfeeding for the recommended duration of time, after their LBW infants have achieved nutritional independence [10].

In their research, Gila-Díaz and colleagues identify that breastfeeding patterns among mothers are largely affected by psychological exposures including depression, stress, and optimism. Fundamentally, the study emphasizes the importance of offering breastfeeding mothers emotional support and social positioning that calms them and supports breastfeeding. Given the importance of breastfeeding adherence in supporting the health of LBW infants, the evidence explored by Gila-Díaz et al. provides a basis for modeling and promoting programs that support breastfeeding [11]. For instance, Mekonnen et al. examine the capacity of the kangaroo mother care intervention program regarding affirming the significance of initiating breastfeeding early. The findings of the study not only affirm the value of breastfeeding programs but also highlight the need to improve breastfeeding practices further to improve health outcomes for LBW infants [12].

## Review

The impact of breastfeeding in preventing infections in infants

Breastfeeding infants during the first six months of life has been proven to be the perfect source of nutrition for infants, as it is a rich source of anti-bacterial and anti-inflammatory bioactive molecules [13]. Understanding the components of human milk can help us to provide a better choice for infant feeding and improve their future health. Human milk contains critical immune factors like secretory immunoglobulins (IgA antibodies), macrophages, T cells, lymphocytes, lactoferrin, and cytokines which protect the child against infections and improve survival [14]. Due to the abundance of immune boosters, breast milk has been shown to possess immunological properties. A study has reported that six weeks of exclusive breastfeeding reduce the incidence of allergic diseases in infants [15]. The findings of a case-control study showed that exclusive breastfeeding during the first six months of life was protective against otitis media while the introduction of formula feeding was tightly linked to a higher risk of acute otitis media [16].

The beneficial role of breastfeeding has also been established in controlling respiratory tract infections which are considered the leading cause of morbidity in children [17]. Pandolfi et al. reported that the protective effects of breastfeeding on respiratory infections extend beyond the first six months, with better outcomes observable up to six years of age [18]. Another study observed that infants who are breastfed have a lower incidence of developing acute respiratory infections (ARI) and diarrhea than infants who are formula fed. It was also found that infants who are breastfed experience shorter ARI episodes and experience a lower percentage of days spent ill compared to infants who were formula fed [19]. Frank et al. found an inverse relationship between exclusive breastfeeding for the first six months and gastrointestinal tract infections, lower respiratory tract infections, upper respiratory tract infections, hospital admissions, acute otitis media, and urinary tract infections (UTIs) [20]. Based on the extensive health-bearing potential of breast milk, the World Health Organization (WHO) has recommended exclusive breastfeeding during the first six months of age [21].

The impact of breastfeeding on weight gain and child development

Infancy provides the best opportunity to improve health outcomes for the later stage of life [22]. Breastfeeding has been described as a protective factor against childhood obesity which often leads to adult obesity. Data from the WHO European Childhood Obesity Surveillance Initiative (COSI) showed that a higher prevalence of obesity is reported in infants who are either raised on formula milk alone or in combination with breastfeeding compared to infants who are exclusively breastfed human milk during the first six months or more. The data was collected from 22 countries, with participants between the ages of six and nine years [23]. Similar findings were reported by Liu et al. who found a significantly reduced risk of obesity in breastfed Chinese children. Their evaluation was based on a survey of 10,753 students between the ages of six to 16 years. The survey revealed that children and adolescents who were breastfed for more than 12 months after birth had significantly lower levels of body mass index (BMI) and obesity [24]. Wang et al. proposed that breastfeeding can impart protective effects against obesity with a relatively lower duration of exclusive breast milk feeding. They demonstrated that breastfeeding even at one month and six months reduces the risk of obesity [25].

However, significant evidence supports that the more exclusive and the longer children are breastfed, the greater their protection from obesity. A meta-analysis of 25 studies showed a dose-response effect between breastfeeding duration and reduced risk of childhood obesity [26]. Although the underlying mechanism of the protective effects of breastfeeding on obesity is unclear, multiple factors seem to influence the positive outcomes of obesity disorder. One key aspect identified in the reduced risk of obesity in breastfeeding children is the lower level of adiposity by human milk feeding compared to formula milk. Gianni et al. examined the effects of a human milk-based diet on the lean and fat mass in late preterm newborns and discovered that exclusive consumption of human milk is associated with decreased deposition of adipose tissues [27]. Higher levels of adipose tissues during early age are linked with negative health-related outcomes including an increased risk for obesity [28].

A significant effort was made by Bartok and Ventura to uncover mechanisms underlying the association between breastfeeding and obesity. The main protective features identified in their investigations were healthy feeding behaviors during infancy, bioactive factors such as immunoglobulins, pituitary hormones, brain-gut peptides, etc. in human milk that regulate growth, and higher quality of weight gain during infancy [29]. Similar views were expressed by Moreno et al. who shared that breastfed babies are more likely to regulate their food intake effectively. They also propagated that due to the nutritious enriched nature of human milk, breastfed babies do not require solid food intake in the first six months of their lives which is linked with a higher risk of obesity [30]. Metzger and McDade demonstrated an association of breastfeeding with the reduced risk of obesity in the siblings model. The data collected from 488 sibling pairs showed that in sibling pairs that had only one breastfed sibling, the BMI of a breastfed sibling was lower compared to his or her sibling [30].

Although exclusive breastfeeding has been linked with slower weight gain in premature infants, better recovery of body composition along with low-fat mass is reported with breast milk feeding. Beliaeva et al. reported that formula-fed premature infants had higher fat mass compared to exclusive breastfeeding infants [31]. A review of literature that analyzed preterm infants’ growth and body composition found that despite the slow early weight gain, maternal breast milk feeding results in better metabolic and neurological outcomes [32]. One study showed that breastfeeding late and preterm infants results in lower fat mass and greater lean body mass deposition and found that lean mass index increases up to three months corrected age compared to formula or mixed-fed infants. This finding strongly supports the use of breastfeeding in the LBW population [33]. Mól et al. also reported that very LBW infants had similar body composition as full-term infants while formula-fed neonates had a higher number of adipose tissue accumulation [34]. Breastfeeding has been associated with a reduced risk of morbidity and mortality in preterm infants due to its protective effects against several diseases. Phukan et al. reported that timely breastfeeding initiation within the first 28 days is associated with improved child survival rates, including all-cause mortality [35].

Ware et al. investigated the role of breastfeeding on infant mortality in Shelby County, Tennessee. The study analyzed the data of 148,679 live births from 2004 to 2014. Their findings showed that breastfeeding initiation is significantly associated with a reduced risk of overall neonatal mortality and infection-related mortality [36]. A multi-center study in China evaluated the role of breast milk on growth patterns and the risk of infections compared to formula-fed premature infants. The participants were divided into two groups based on their feeding regimen, i.e., over 50% human breast milk and formula milk. Although both groups showed similar growth patterns, human milk-fed infants had a lower occurrence of sinus infections and feeding tolerance. Among the 125 cases included, 62 cases were breast milk and 63 cases were formula milk powder. There was no significant difference in gestational age, head circumference, length of birth, length of hospital stays, and weight gain rate. Nosocomial infections were significantly less common in the breastfed group than in the preterm infant-fed group [37]. Yu et al. compared the effects of breast milk and formula on very LBW infants and found that breastfed infants had a lower risk of necrotizing enterocolitis (NEC) and shorter hospital stays than formula-fed infants. On the other hand, those who were formula-fed gained more weight and length. However, there were no differences between the two groups in terms of head circumference, incidence or progression of sepsis, the incidence of retinopathy of prematurity (ROP), and mortality [38].

A 2018 study of 1,809 participants compared the effects of breastfeeding versus formula feeding on the growth of premature or LBW infants. The findings revealed that the formula-fed group experienced faster rates of weight gain, linear growth, and head growth. The infants in the formula group had a higher risk of NEC than the breastfed group. None of the studies assessing neurodevelopment after childhood produced significant results, and there were no differences in psychomotor or intellectual development between the two groups. There was also no difference between the two groups in terms of exposure to invasive infection [39]. Similar results were reported by McGuire and Anthony who analyzed differences between breastfeeding and formula feeding. They reported that infant formula promotes faster, shorter-term growth velocities in LBW neonates compared to breastfed infants. There is not enough information to draw firm conclusions about whether adverse effects, such as NEC, are common in newborns who receive formula milk instead of breast milk [40]. Corpeleijn et al. evaluated the effect of breast milk versus formula milk on reducing the incidence of severe infection, NEC, and mortality in the first 10 days of life in 373 infants (183 breast milk, 190 formula). No significant effect of donated human milk on the prevention of major infections, NEC, and all-cause mortality in the first 10 days of life was found in preterm infants [41].

NEC is widely recognized as a devastating intestinal disease for very low birth weight infants (VLBW) [42]. A review of 32 studies by Altobelli et al. reported that breastfeeding reduces the risk of developing NEC in infants. Preterm babies with LBW who were breastfed showed significantly less incidence of NEC as compared to formula-fed infants [43]. Quigley et al. found that breastfeeding was associated with reduced hospital visits related to diarrheal and respiratory tract infections during the first eight months of life [44]. Levy et al. evaluated the relationship between UTIs and breastfeeding in preterm infants (less than 37 weeks gestation period). The finding described that breastfeeding during early age is linked with a lower risk of UTIs in infants. The protective effects can be attributed to the high concentrations of immunoglobulins in breast milk that inhibits bacteria from binding to gut mucosa [45]. A recent systemic review and meta-analysis by the American Academy of Pediatrics found no significant differences in mortality, infection, cognitive development, and growth between breastfed and formula-fed infants. However, formula milk was associated with a higher risk of NEC in infants. The investigators reported a high level of uncertainty regarding the evidence in studies and highlighted the need to improve the quality of such observation studies, so the true effect on prognosis can be determined [46].

Breast milk is considered the highest nutrition source for the infant and is superior to formula milk in composition. It contains highly nutritional components like fat which is very important to the infant as it provides energy and is essential for the nervous system development. Protein is predominant in both formula milk and human milk composition. There are two types of protein in milk i.e., casein and whey [47]. Whey protein is easier to digest than casein. As formula milk has a high amount of casein protein, it makes it more difficult to digest in the infant’s stomach [48]. Breast milk has an adequate number of vitamins except for vitamin D and Vitamin K. Infants who are maintained exclusively on breastfeeding can lack optimum levels of vitamin D which put them at risk for developing vitamin D deficiency compared to infants who are on formula feeding.

An advantage of formula milk compared to human milk is that there are different types and options that help in providing adequate nutrition to a variety of infants [49]. Infant formulas fall into three categories i.e., made with cow's milk, soy-based, and others made with unique ingredients. They differ in terms of price, taste, digestion, nutrition, and calories. There are various types of formulations available to suit different demands. Some alternatives to cow's milk are based on amino acids or contain a significant amount of hydrolyzed whey or casein proteins. Some formulas are made with rice [48]. According to a cross-sectional study, newborns ingest more sugar intake every day than toddlers. The study also showed that added sugars from all sources, varied by age group, were strongly connected to rapid weight gain. The most of additional sugars taken by toddlers came from dining meals, whereas the majority of sugars taken by newborns came from a formula. The majority of formula-fed and breastfed participants had similar mean calorie intakes, but the formula-fed received more sugar intake from all sources during the supplemental feeding phase. Early dietary experiences mold adult food preferences and reduce the likelihood of obesity [50]. Breastfeeding provides lifelong protection against obesity as it provides moderate levels of sugar. A study of 32,000 children found that breastfed children had a significantly lower risk of obesity than formula-fed children [51].

## Conclusions

Breast milk is the primary source of optimum nutrition in most infants. Significant research has shown that human milk is superior in composition compared to any other available alternative. It is enriched with fats, protein, carbohydrates, and several biologically active compounds such as immunoglobulins, lactoferrin, and lymphocytes. These compounds impart several health benefits that are often unavailable from formula feeding. Breastfeeding for the first six months is of vital importance as it can be a measure of future health. The WHO and UNICEF recommend initiating breastfeeding within the first of birth and continuing exclusive breastfeeding for a least six months. Exclusive breastfeeding for the first six months has protective effects against gastrointestinal tract infections, lower respiratory tract infections, upper respiratory tract infections, hospital admissions, acute otitis media, and UTIs. Breast milk feeding has been attributed to non-fat mass accumulation which reduces the risk of childhood obesity. Although formula milk feeding has shown a higher growth rate in infants, breast milk showed a better recovery of body composition along with low-fat mass accumulation. Although breast milk showed improved resistance against disease, there was no significant difference in mortality compared to formula-fed milk. In conclusion, breast milk is an optimal source to fulfill nutrition in premature, LBW infants and provide protective effects against future disease challenges.
